# Entropy convergence in early bilinguals’ syntactic packaging

**DOI:** 10.3389/fpsyg.2022.1010002

**Published:** 2022-10-14

**Authors:** Helen Engemann

**Affiliations:** Department of English Linguistics, University of Mannheim, Mannheim, Germany

**Keywords:** bilingual first language acquisition, syntactic packaging, variability, entropy, regularization, convergence, motion events, English and French

## Abstract

A core question in developmental and cognitive research concerns the way linguistic variation affects the acquisition process. Previous research on monolinguals suggests that children, but not adults, tend to regularize inconsistent input, resulting in reduced variation. Some recent claims explain regularization as a general bias linked to cognitive load. However, little is known about bilingual acquisition contexts where children naturally experience both increased variability and cognitive load. This study investigated the impact of between- and within-language variability in syntactic packaging (i.e., how semantic elements are mapped onto syntactic units) on simultaneous bilinguals’ acquisition of motion event encoding. In this domain, French is considered highly variable, in contrast to low variability demonstrated by English. Based on this crosslinguistic contrast, 96 English–French bilingual children (aged 4–11 years) and 96 age-matched monolinguals of each language described 32 animated cartoons showing complex motion events. Children’s variability of selected syntactic patterns was measured using the information-theoretical concept of entropy. Results indicated that bilingual children significantly reduced syntactic variation relative to monolingual peers, but only in French, the more variable language. Moreover, bilingual children converged in entropy levels across the two languages and patterned mid-way between respective monolinguals. These findings suggest that the cognitive load inherent in bilingualism is not sufficient to explain reduced linguistic variation. Instead, the asymmetric drop in entropy highlights the strong impact of crosslinguistic differences and thus underlines the importance of taking language-specific factors into account in theories of cognitive load.

## Introduction

How do bilingual children deal with linguistic variation? A well-known finding from cognitive developmental research (e.g., Hudson Kam and Newport, 2009; Austin et al., 2022) is that children regularize unpredictable variation, that is, they impose structure on “noisy” input by systematizing the use of a dominant variant, whereas adults reproduce irregularities, a behavior referred to as probability matching. The reason for this difference between children and adults is still an open question. Children’s limited cognitive capacities (e.g., memory) have been suggested as potential cause (Hudson Kam and Newport, 2009). Indeed, some studies have found regularization in adults when put under conditions of cognitive load (Hudson Kam and Newport, 2009; Fehér et al., 2016; Ferdinand et al., 2019), resulting in a similar reduction of unpredictable variation in speakers’ output (but see Perfors, 2012).

While regularization has been mainly investigated either with monolingual speakers in laboratory contexts using artificial learning paradigms or in the context of creole formation (Sankoff, 1979; Bickerton, 1984), it has so far been scarcely addressed in naturalistic situations of child bilingual development. These inherently involve more input variability as children are exposed both to variation within each language and across the two language systems. In addition, bilingual children often hear at least one of their languages from non-native sources, e.g., when a caregiver speaks one of the languages as a second language to communicate with the other caregiver, thus adding to the overall input variation. This makes bilingual language acquisition a particularly relevant and insightful window into the impact of within- and between-language variability on acquisition. If regularization is indeed a more general tendency linked to cognitive pressure (e.g., Ferdinand et al., 2019), this raises the question of whether bilingual children might show a more prominent drive to reduce variation than monolinguals. For first, acquiring and using two languages is often claimed to entail greater cognitive load (e.g., Silva-Corvalán, 1994; Polinsky and Scontras, 2020; Adamou, 2021) owing to the constant need to manage cross-language competition. Furthermore, greater variability entailed by dual language settings may result in greater pressure to reduce it to free up processing resources (e.g., Sorace, 2011; Sequeros-Valle et al., 2020).

Although there is hardly any research explicitly addressing the impact of variability in bilingual acquisition contexts, some studies have observed bilingual tendencies to curtail morphosyntactic variation. For example, a study on early Dutch-German bilinguals showed reduced variation in the use of progressive aspectual markers compared to monolinguals (Flecken, 2011), manifesting as an overextension of the most frequent form to semantically constrained contexts, at the expense of less frequent alternatives. Similarly, Spanish–English bilingual children have been reported to reduce variation in the Spanish modal system by overextending the indicative to contexts in which monolinguals show more variable subjunctive vs. indicative usage (Silva-Corvalán, 1994). Hendricks et al. (2018) investigated how inconsistent input in an endangered minority language context affects the acquisition of gender marking in the North-Frisian dialect of Faring. Findings demonstrated that only children with sufficient regular exposure to Faring approximated adult-like probability patterns, while all others regularized. As bilingual exposure in many cases implies quantitatively reduced input from each language relative to monolinguals, these findings raise important questions about whether variability might affect bilingual acquisition settings more strongly or differently than monolinguals.

The present study extends existing morphosyntactic research to the acquisition of form-function mappings in the domain of motion event expression, a referential domain characterized by crosslinguistic as well as intra-linguistic variability. The former is captured by Talmy’s (2000) typological distinction between satellite- and verb-framed languages, based on whether speakers typically encode path (the core schema of motion) in a verb root (e.g., *il sort*, ‘he exits’) or in so-called satellites, such as particles or prepositional phrases (e.g., *into the room*). The extent to which these framing tendencies are followed by speakers varies across languages. Verb-framing languages in particular have been observed to exhibit considerable variation in framing choices. Some authors have therefore argued for their reclassification as split or hybrid systems (e.g., for French: Kopecka, 2006; for Italian: Iacobini and Masini, 2006). An innovative recent approach by Montero-Melis (2021) has attempted to quantitatively capture such variability in terms of the information-theoretical concept of entropy (see Cover and Thomas, 2005), representing the degree of “disorder” or unpredictability in a dataset. Using this approach, Montero-Melis (2021) revealed striking crosslinguistic differences in speakers’ consistency of framing selection (high entropy values in Spanish vs. low values in Swedish).

Variability in motion expression also extends to the syntactic packaging patterns (i.e., the mapping of semantic elements onto syntactic units) that speakers of different languages use to combine the expression of several event components (e.g., manner and path). Thus, speakers of verb-framing languages tend to resort to syntactically more complex structures, using embedded clauses (such as gerundive constructions, e.g., *Elle traverse la rue en courant*, ‘She crosses the road running’). Another tendency in verb-framing languages consists in relying more on parataxis; thus, speakers spread information across several independent clauses (e.g., *Elle traverse et elle court*, ‘She crosses and she runs’). Both strategies are uncommon in satellite-framing languages, where speakers instead consistently choose to package information tightly within syntactically simple clauses by using satellites to communicate path information, thus freeing up the verb slot for manner (*She ran across the road*). While this pattern is also available to some extent in French and other verb-framing languages (e.g., *Elle court jusqu’à l’autre côté*, ‘She runs to the other side’), it is semantically constrained and hence cannot be applied systematically. Thus, when communicating more than one event component, there seems to be considerable crosslinguistic difference with respect to the variety of syntactic constructions that speakers habitually employ. Developmental research suggests that these crosslinguistic differences in syntactic packaging preferences affect monolingual first language acquisition, such that children mirror adult target patterns from early on (Berman and Slobin, 1994; Allen et al., 2007; Berman and Nir-Sagiv, 2008; Hickmann et al., 2018). Although there are some studies on early bilingualism investigating the effects of cross linguistic differences on semantic encoding (Miller et al., 2018; Engemann, 2022), bilingual acquisition research has so far not looked at syntactic variability in motion events as a phenomenon of interest.

The aim of this study is to examine the impact of variation in simultaneous English-French bilingual children’s syntactic packaging patterns when describing caused motion events by comparing variability levels across the two languages as well as across the two acquisition settings (monolinguals vs. bilinguals) in order to address the following research questions: (i) Does syntactic variability in caused motion encoding differ between bilinguals and monolinguals? (ii) What is the impact of language on variability? Following the lead of Montero-Melis (2021), variability will be operationalized as entropy (for details, see “Entropy computation”). This has several advantages. First, entropy has proven a precise concept allowing quantification of variability (see also Gullifer and Titone, 2020 for entropy as quantification of variability in bilinguals’ language experience), which is also suitable for categorical variables and is at the same time flexible enough to allow for meaningful crosslinguistic comparison (Montero-Melis, 2021, p. 6). Second, entropy reduction has been successfully used in past research as a measure to identify and operationalize regularization (Ferdinand et al., 2019). In line with this latter line of research, regularization will be defined as reduction in entropy.

We formulate the following hypotheses. Regarding language effects, entropy levels are expected to be higher in French than in English, independently of acquisition setting, in line with previous work qualitatively suggesting more variability in verb-framing than in satellite-framing languages. As for bilingual effects, several potential outcomes are entertained, based on considerations of cognitive load:

- Bilinguals mirror monolinguals in each of their languages, i.e., entropy levels do not differ between acquisition settings.
- Bilinguals reduce entropy irrespective of language, i.e., they show decreased levels when compared to monolinguals of each language.
- Bilinguals selectively reduce entropy in accordance with language-specific pressures, i.e., more, or exclusively in French, but less or not at all in English.

Thus, scenario B would support a view of entropy reduction as a potentially more general bilingual-specific tendency, while scenario C would favor an account emphasizing the role of language-specific factors.

## Materials and methods

### Participants

A total of 192 children between 4 and 11 years (*M* = 7.4, SD = 2.2; 95 females) participated in this study. There were 96 simultaneous bilinguals who were raised hearing both French and English from birth and another 96 monolingual children, half of them English (*n* = 48), and half French (*n* = 48). Following a between-subjects design, bilingual children were randomly assigned to one of two language groups (*n* = 48/group), one of which performed the experiment in English (henceforth referred to as “English bilinguals”), the other one in French (henceforth “French bilinguals”), to avoid undesirable practice and priming effects between languages. The combination of language of testing and acquisition setting (bilingual vs. monolingual) yielded four groups of 48 children each: English monolinguals (*M* = 7.0, SD = 2.3; 23 females), French monolinguals (*M* = 7.4, SD = 2.3; 24 females), English bilinguals (*M* = 7.4, SD = 2.1; 24 females), and French bilinguals (*M* = 7.8, SD = 2.2; 24 females).

Bilingual participants were recruited from daycares and primary schools in France practicing dual-language immersion, to ensure regular and balanced input from both languages both at home and at school/daycare. To control for effects of the societal language on motion expression (Daller et al., 2011), bilingual participants were recruited and tested in France. Monolingual French children were raised and tested in Paris (France), monolingual English children in Cambridge (United Kingdom). Caregivers of all participating children filled out language background questionnaires about exposure patterns and family language practices. In the case of monolinguals, children who received regular naturalistic exposure to any other language than their L1 were excluded from the sample. In addition, in the case of bilinguals, caregivers’ ratings of proficiency levels (ranging from 1 = ‘poor’ to 10 = ‘native-like’ on a Likert scale) served to exclude all participants whose ratings diverged by more than two points between their two languages. This was to ensure that bilinguals were maximally balanced and to thus minimize the risk of imbalanced proficiency as a cause for any deviations in entropy scores.

### Materials

**Figure 1:**
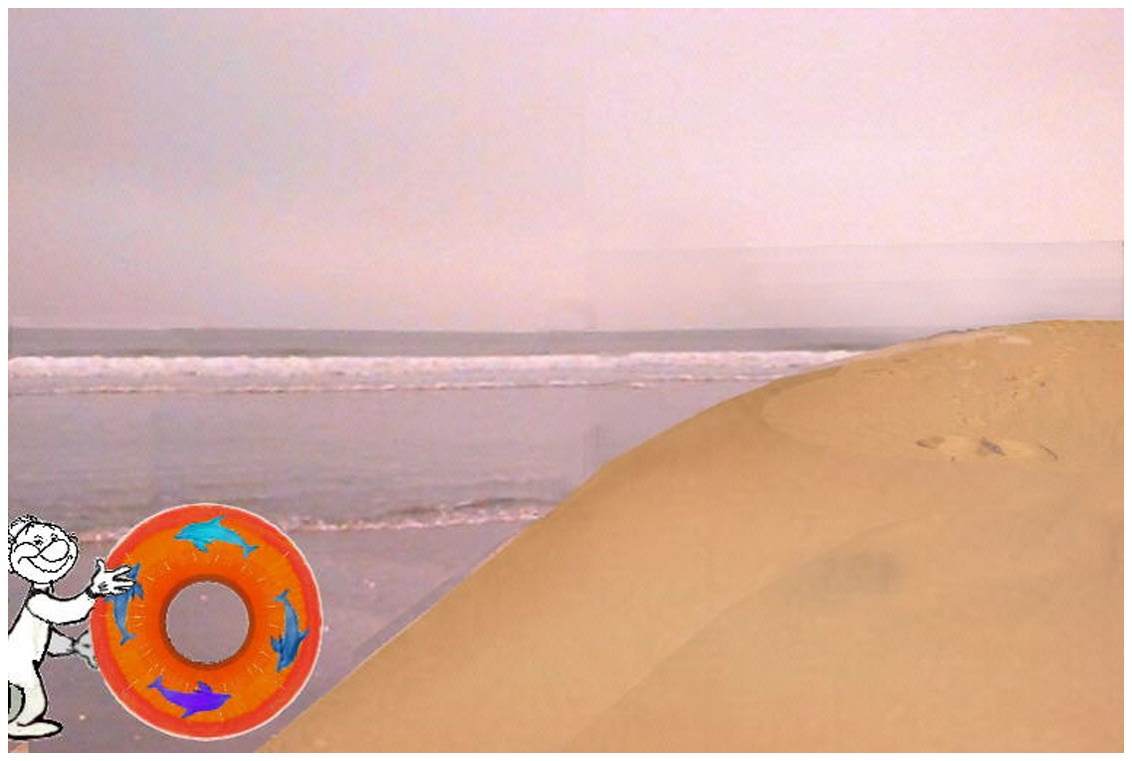
Still image of a target video used in the study.

A total of 32 short video animations (Hickmann and Hendriks, 2010) were used to elicit caused motion descriptions. Target items showed a human character causing an object to move (e.g., by rolling a swim ring up a sand dune) along a given path and in a certain manner. An example is provided in Figure 1. Each video portrayed 5 motion-relevant semantic components that could be selected for expression (see supplementary Table A), 3 of which were manipulated across items (4 × path, 2 × causing manner, and 2 × object’s manner), while 2 were held constant (cause and agent’s manner of motion). Seven filler items occurred regularly at predefined intervals. Items were presented in four semi-randomized orders to which children were randomly assigned.

### Procedure

Children were tested individually in a quiet space at their school. They were instructed to watch each video carefully one by one on a laptop screen and to report what had happened after the end of each item for the sake of a fictitious listener who could not see the videos. For children up to age 5 the listener was a doll they were presented to at the beginning of the session and who was then blindfolded and seated with her back facing the screen. To minimize crosslinguistic influence in the case of bilingual children, the experimenter induced a monolingual mode by initiating a conversation in the language of testing a few minutes before the start of the task. All sessions were audio-recorded and transcribed according to CHAT conventions (MacWhinney, 2000).

### Coding

Target motion descriptions were coded along two syntactic dimensions: (i) subordination, that is, the presence or absence of minimally one subordinate clause (coded as ‘complex’ vs. ‘simple’), and (ii) compactness, distinguishing ‘tight’ vs. ‘loose’ packaging. When information was packaged within either a single main clause [see (1)–(2)] or a main matrix clause with one or several subordinate clauses [see (3)–(4)], the response was treated as ‘tight’, whereas responses spreading information across several syntactically independent clauses were coded as ‘loose’ [see (5)–(8)]. Crossing the two syntactic dimensions of subordination and compactness thus produced the following four possible values of syntactic packaging patterns: ‘TS’ (tight simple): Information is expressed in one simple compact clause [see (1)–(2)]; ‘TC’ (tight complex): Information is expressed in one sentence, containing at least one subordinate clause [see (3)–(4)]; ‘LS’ (loose simple): Information is distributed across two or more coordinated [see (5)–(6)] or juxtaposed [see (7)–(8)] clauses; and ‘LC’ (loose complex): Information is distributed across multiple main clauses, just as in LS, but containing at least one subordinate clause, as shown in (9) and (10).

1. He rolls the ball across the road.
2. Il pousse le ballon jusqu’en haut de la colline.
  ‘He pushes the ball all the way to the top of the hill.’
3. He crossed the road walking while rolling the ball.
4. Il monte la colline en poussant le ballon.
  ‘He ascends the hill by pushing the ball.’
5. He’s crossing the road and he’s rolling the ball.
6. Il monte la colline et il fait rouler le ballon.
  ‘He ascends the hill, and he rolls the ball.’
7. He’s crossing the road. He’s also rolling a ball.
8. Il monte la colline. Il pousse le ballon.
  ‘He ascends the hill. He pushes the ball.’
9. He was rolling the ball. He walked across the road pushing it.
10. Il est en train de marcher et il monte en poussant le ballon.
  ‘He’s walking and he ascends pushing the ball.’

### Entropy computation

To provide a measure of the variability of syntactic packaging patterns, entropy scores were computed based on Shannon’s original information-theoretic formula (Shannon, 1948), measuring the degree of uncertainty in a variable:


$$H\left(\mathrm{Syntactic Packaging}\right)=-\underset{x\in X}{\sum }p\left(x\right)\log_{2}p\left(x\right)\text{.}$$

Entropy was computed by participant following the procedure of Montero-Melis (2021), using the categorical variable of syntactic packaging with four possible values (TS, LS, TC, and LC). The entropy of syntactic framing was thus calculated based on the probability (*p*) of each of the four values (*x*) that syntactic packaging can assume (*p*(*x*)). A high entropy score *H*(syntactic packaging) means that the choice of syntactic packaging patterns is unpredictable (highly variable), whereas low entropy scores indicate that packaging choices are more predictable (low variability). Thus, children selecting each of the four syntactic packaging patterns with similar likelihood will receive a high entropy score (put differently, their choice of pattern is highly unpredictable), whereas children who show a consistent preference for a specific pattern will have a low entropy score.

## Results

In what follows, descriptive statistics are first reported for syntactic packaging patterns (TS, LS, TC, and LC) and entropy scores averaged across the four examined groups, followed by the inferential analysis testing for group differences in terms of entropy.

### Syntactic packaging patterns

**Figure 2:**
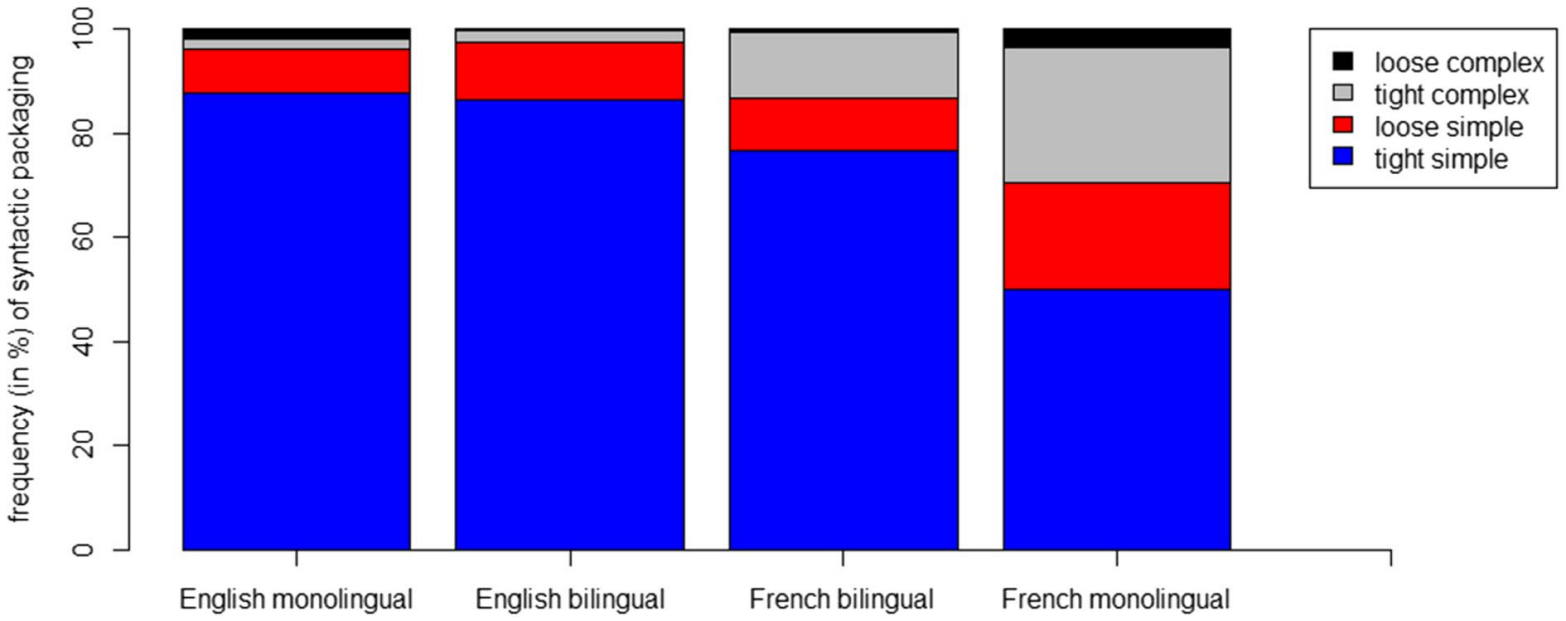
Frequencies (in %) of syntactic packaging choices by language acquisition type.

Figure 2 shows the proportions of each of the four syntactic packaging patterns selected in children’s event descriptions as a function of language acquisition type. Visual inspection reveals that tight simple structures constitute by far the most frequent packaging category across all groups. Recall that this pattern corresponds to target responses that express the motion event within one single main clause [see (1) and (2) above]. Nonetheless, tight simple responses occur much more rarely in French monolinguals (50%) in comparison to English monolinguals (88%) and bilinguals (87%), but also relative to French bilinguals (77%). In contrast, French monolinguals exhibit a much more variable pattern, making more use of tight complex (26%) as well as loose simple (20%) patterns, both of which represent relatively marginal categories in the other groups. Furthermore, it is interesting to observe that while bilinguals’ English responses align closely with those of English monolinguals, bilinguals’ French descriptions diverge starkly from those of their monolingual counterparts and instead show a mid-way pattern between English and French monolinguals. In summary, the descriptive analysis of the data suggests that the variability of syntactic packaging choices is strongest in French monolinguals and conversely, lowest in English monolinguals and bilinguals, while French bilinguals occupy an in-between position.

### Entropy

**Figure 3:**
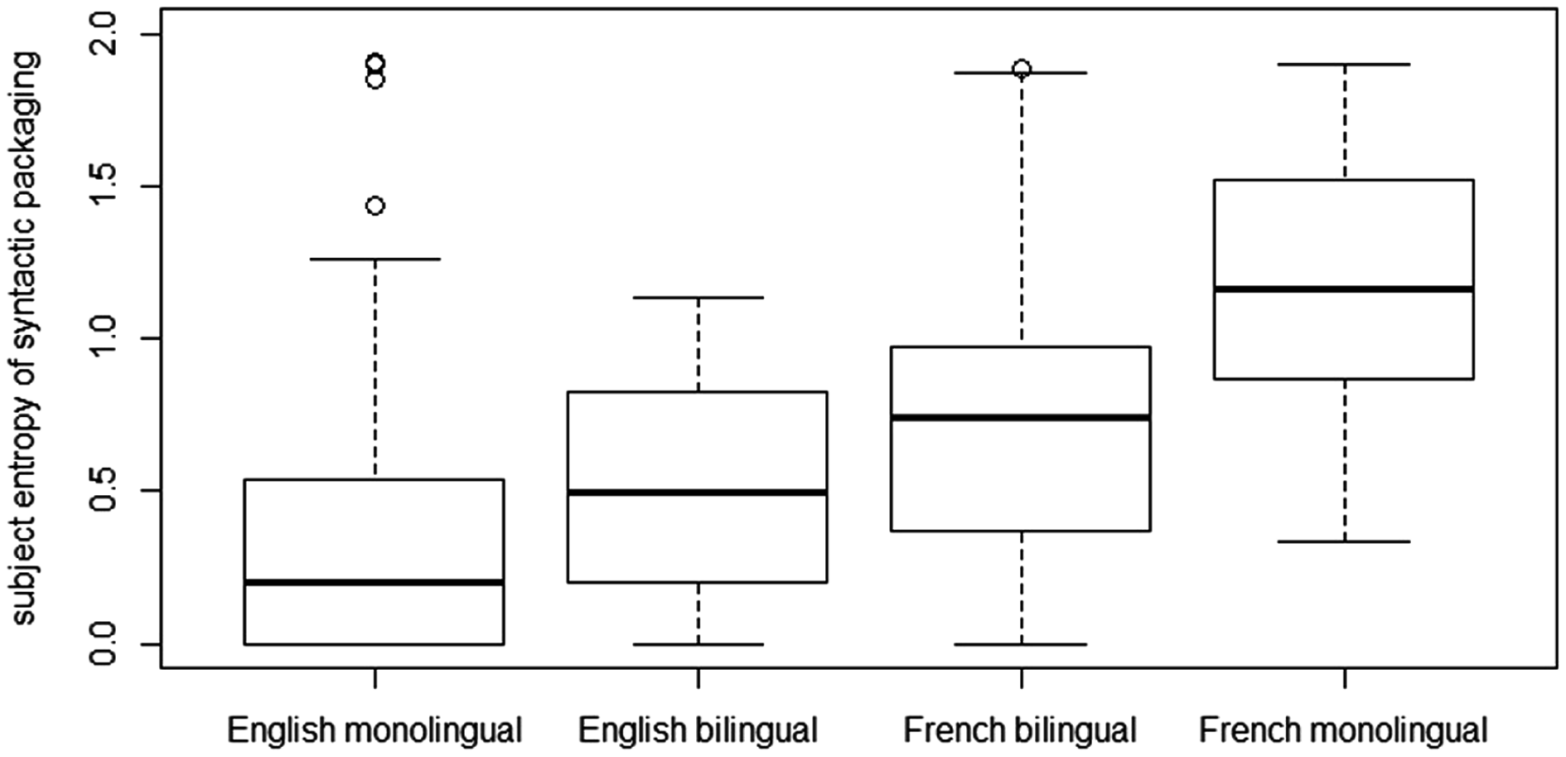
Spread of participants’ entropy scores as a function of language acquisition type (median and interquartile range).

Entropy scores were calculated for each participant based on children’s use of the four possible syntactic packaging patterns. The boxplot in Figure 3 illustrates the distribution of entropy scores by group. Visual inspection supports the picture obtained from overall proportions reported above (see Figure 2). Thus, syntactic entropy is highest in monolingual French descriptions (*M* = 1.17; SD = 0.36): and lowest in the monolingual English data (*M* = 0.39; SD = 0.48), while bilingual children of both languages pattern in between the extremes of respective monolinguals. It is noteworthy that French bilinguals’ entropy scores (*M* = 0.73; SD = 0.44) are further removed from their monolingual counterparts’ than English bilinguals (*M* = 0.52; SD = 0.35) are from those of corresponding English monolinguals. Overall, bilingual French descriptions appear to align more closely with English than with French monolinguals.

A Kruskal–Wallis rank sum test (since the data was not normally distributed) was run on entropy scores with language acquisition as independent variable to address the question of whether groups differed with respect to syntactic entropy. The analysis revealed that language acquisition type significantly affected entropy scores (*χ*^2^(3) = 66.4, *p* < 0.001). *Post-hoc* comparisons using Dunn’s procedure were performed with Bonferroni-adjusted *p*-values. The results, summarized in Table 1, support the picture emerging from Figure 3. First, in accordance with predictions, there were striking crosslinguistic differences indicating significantly higher entropy levels in French than in English. Significant differences were obtained for all crosslinguistic group comparisons. Second, French bilinguals’ entropy scores were significantly lower than those of monolingual French peers (*p* < 0.001), but also significantly higher than those of English monolinguals (*p* < 0.01), thus confirming the intermediate pattern observed earlier (see Figures 2, 3). Notably, while French bilinguals’ entropy scores showed robust differences from both English and French monolinguals, they did not diverge significantly from those of bilingual English children (*p* = 0.32), thus suggesting convergence between the two bilingual groups. English bilinguals, on the other hand, converged statistically with both English monolinguals and French bilinguals. Although the boxplot in Figure 3 suggests that both bilingual groups occupied a mid-level of entropy relative to respective monolinguals, the group comparison between bilinguals and monolinguals did not reach significance in the English data (*p* = 0.44). This confirms the descriptive picture obtained from Figure 2, indicating close alignment between monolinguals and bilinguals in English descriptions. The most noteworthy findings relating to bilingualism thus concern the significantly lower entropy levels in the French bilingual data relative to French monolinguals and the entropy convergence observed between English and French bilinguals.

**Table 1:** Pairwise comparisons between groups concerning entropy levels.

Comparison	*Z*	*p*-value	
English bilingual–English monolingual	1.8	0.436	n.s.
English bilingual–French bilingual	−1.93	0.319	n.s.
English monolingual–French bilingual	−3.73	0.001	^*^
English bilingual–French monolingual	−5.96	< 0.001	^***^
English monolingual–French monolingual	−7.76	< 0.001	^***^
French bilingual–French monolingual	−4.03	< 0.001	^***^

Results are obtained using Dunn’s procedure with Bonferroni-adjusted *p*-values. Significance codes: ‘***’: *p* < 0.001; ‘**’: *p* < 0.01; ‘*’: *p* < 0.05; ‘.’: *p* < 0.1; ‘ns’: *p* > 0.1.

## Discussion

This study investigated how bilingual children deal with syntactic variability, operationalized as entropy, within and between language systems in the domain of motion expression. The hypothesis concerning the impact of language was borne out by the data. Thus, French descriptions overall were characterized by significantly higher entropy levels, that is, greater variability in the choice of syntactic packaging patterns, than descriptions in English. This confirms observations from previous research comparing verb- and satellite-framed languages in this domain in terms of consistency of syntactic framing (e.g., Hickmann and Hendriks, 2010; Montero-Melis, 2021).

The main question addressed by this study was how such crosslinguistic differences in syntactic variability would play out in the context of early bilingualism given assumptions of cognitive load. Our findings in this respect are consistent with scenario C, predicting asymmetric entropy reduction between French and English as a result of language-specific differences. Thus, bilingual children’s descriptions did reveal a significant reduction in syntactic variability relative to monolinguals, but importantly, this reduction was limited to French, i.e., the language characterized by strong variability in the domain of motion. In contrast, variability levels in bilinguals’ English descriptions did not significantly deviate from English monolinguals. This finding speaks to the importance of considering language-specific factors in gauging the cognitive costs entailed by variability in a given domain. Thus, wholesale reduction of syntactic variability is not a necessary outcome of bilingualism, despite the presumed cognitive pressure arising from the added overall variation incurred by dual language exposure.

The finding of converging entropy levels in the two bilingual groups proves particularly interesting in this respect: While there was a clear entropy decrease in bilinguals’ French, there was also a trend (albeit not significant) suggesting a slight boost in entropy in English bilinguals compared to monolinguals. If the cognitive load of bilingualism were responsible for decreasing entropy across the board, it clearly cannot explain this opposite trend in our English data. In turn, such selective drop in entropy suggests that when language-specific baseline levels of entropy are already low, as they are in English motion expressions, crosslinguistic influence from the other language may afford the introduction of innovative variants into the less variable language and may hence also potentially boost the range of syntactic patterns used. A qualitative look at the English data did in fact reveal bilinguals’ occasional use of idiosyncratic participial structures (e.g., *he crossed by pulling it*), which appear to be modelled on French gerundive constructions (*en tirant*, ‘by pulling’). The fact that these never occur in monolinguals’ English descriptions supports an analysis in terms of a structural innovation from French, boosting structural variability within English. Such bilingual increase in variation would be in line with what has been found in other linguistic domains, such as phonology (e.g., see Levy and Hanulíková, 2019 for vowel production).

There is another interesting possibility suggested by bilingual children’s converging entropy scores. Overall, the picture that arises is that of bilingual children converging somewhere in between the two monolingual extremes. Particularly in the case of French bilinguals, it is noteworthy that quantitatively speaking, children’s average entropy scores are almost exactly located mid-way between English and French monolinguals, thus indicating a statistical sensitivity towards the probabilistic patterns present in the input of both of their languages. Based on this, an alternative account of the data could amount to bilingual children probability-matching across their two languages, such that children track the statistical probabilities of syntactic packaging in English and French. There is some evidence from existing research that children are sensitive to statistical patterns in their linguistic environment (e.g., Schuler et al., 2016 for morphosyntax in 5- to 8-year-olds). Settling on a quantitative mid-point would be a highly cost-efficient strategy allowing children to effectively reduce the cost of probability matching in both of their languages separately. Depending on how far entropy scores diverge between the two languages involved, such tendency would also be compatible with the finding of increases as well as decreases in entropy in each language relative to monolinguals. In other words, it is not the case that bilingualism *per se* can be expected to necessarily lead to a decrease in variability; instead, converging mid-way could involve a decrease in one language and an increase in the other, or alternatively, no deviations from monolinguals in cases in which the two languages exhibit similar levels of variability.

To conclude, the present findings call for a reappraisal of the construct of cognitive load in the context of bilingualism. Specifically, I propose that bilingualism should not automatically be equated with increased cognitive load giving rise to entropy reduction without due consideration of the language-specific properties of each language and their differential degrees of variability in the domain under investigation. Consequently, the extent to which production patterns are affected by entropy reduction (or boost) in a specific bilingual constellation may differ substantially in different language pairs and may also manifest differently in each language.

For future research, there are several testable predictions that can be generated from this proposal. If language-specific levels of variability indeed play a role for overall cognitive load, then we should find more crosslinguistically symmetrical levels of regularization in domains in which both languages feature comparable degrees of variability. In this respect, it would be illuminating to compare the present findings with bilingual contexts of two syntactically highly variable verb-framing systems, such as French and Spanish.

A limitation of the present study is the lack of adult control data, which would allow us to disentangle developmental from language-specific factors. It would therefore be important to explore syntactic variability from a developmental perspective to investigate the extent to which monolingual as well as bilingual children, adolescents and adults regularize or probability-match. Recent research on monolinguals suggests that regularization of noisy input is modulated by maturational and cognitive capacities (Austin et al., 2022). The fact that in the present study, entropy convergence is observed in children as old as eleven years raises the question whether these effects phase out or persist into adolescence and adulthood. Future research should therefore test whether bilingual adolescents and adults show similar asymmetric divergences from monolingual adult speakers in their motion expressions or whether they gradually come to match language-specific variability levels in each of their languages.

## Data availability statement

The raw data supporting the conclusions of this article will be made available by the authors, without undue reservation.

## Ethics statement

The studies involving human participants were reviewed and approved by the University Research Ethics Committee, University of Cambridge, United Kingdom. Written informed consent to participate in this study was provided by the participants' legal guardian/next of kin.

## Author contributions

The author confirms being the sole contributor of this work and has approved it for publication.

## Funding

The publication of this article was funded by the University of Mannheim.

## Conflict of interest

The author declares that the research was conducted in the absence of any commercial or financial relationships that could be construed as a potential conflict of interest.

## Publisher’s note

All claims expressed in this article are solely those of the authors and do not necessarily represent those of their affiliated organizations, or those of the publisher, the editors and the reviewers. Any product that may be evaluated in this article, or claim that may be made by its manufacturer, is not guaranteed or endorsed by the publisher.
