# Supplementary material for: Entropy convergence in early bilinguals’ syntactic packaging
Source: Front Psychol. 2022 Oct 14;13:1010002. doi: 10.3389/fpsyg.2022.1010002 (PMC9616223; doi:10.3389/fpsyg.2022.1010002)
Supplement: Supplementary file 1 [file Table_1.pdf]

Supplementary Materials

Table A. Semantic components portrayed in target items

Semantic component	Presentation in stimuli
Causal relation between agent and object	constant: agent causes object to move
Agent's Manner of motion	WALK
Causing Manner (agent's action causing displacement)	PUSH, PULL
Object's Manner of motion	ROLL, SLIDE
Path (trajectory followed by agent and object)	UP, DOWN, ACROSS, INTO
